# Supplementary material for: Group II truncated haemoglobin YjbI prevents reactive oxygen species-induced protein aggregation in Bacillus subtilis
Source: eLife. 2022 Sep 20;11:e70467. doi: 10.7554/eLife.70467 (PMC9536834; doi:10.7554/eLife.70467)

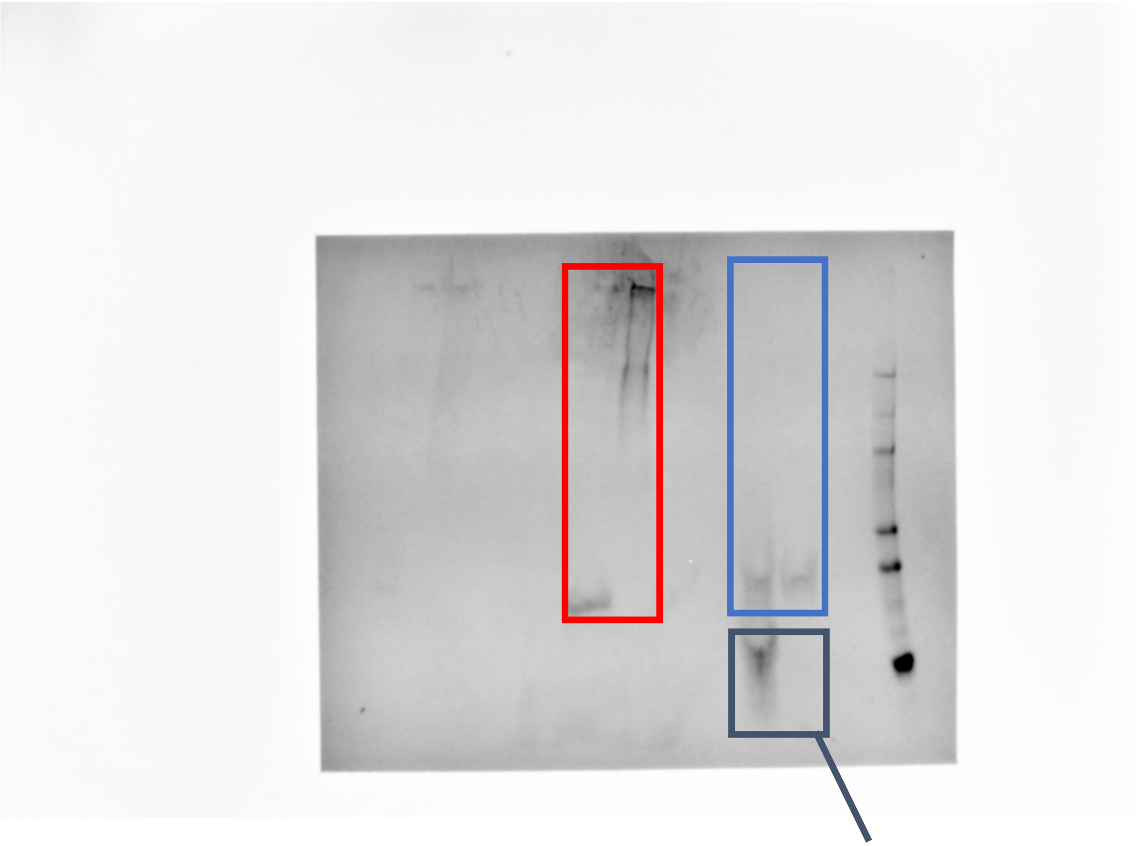
Non-reproducible area

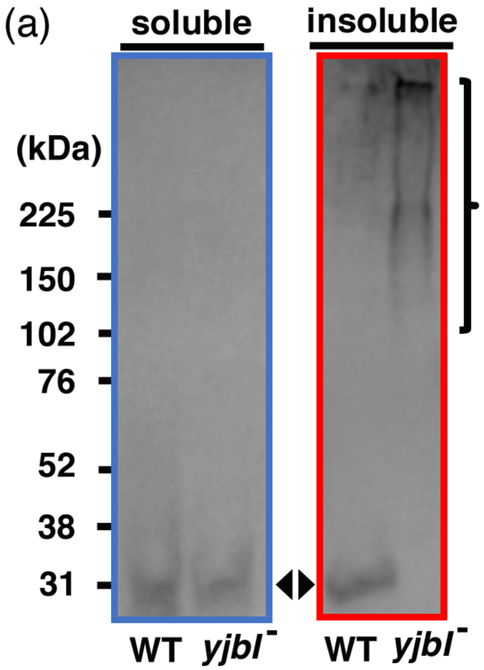
(a)
soluble
insoluble
(kDa)
225
150
102
76
52
38
31
WT
yjbI-
WT
yjbI-

Reproducibility of result with independent soluble fraction samples

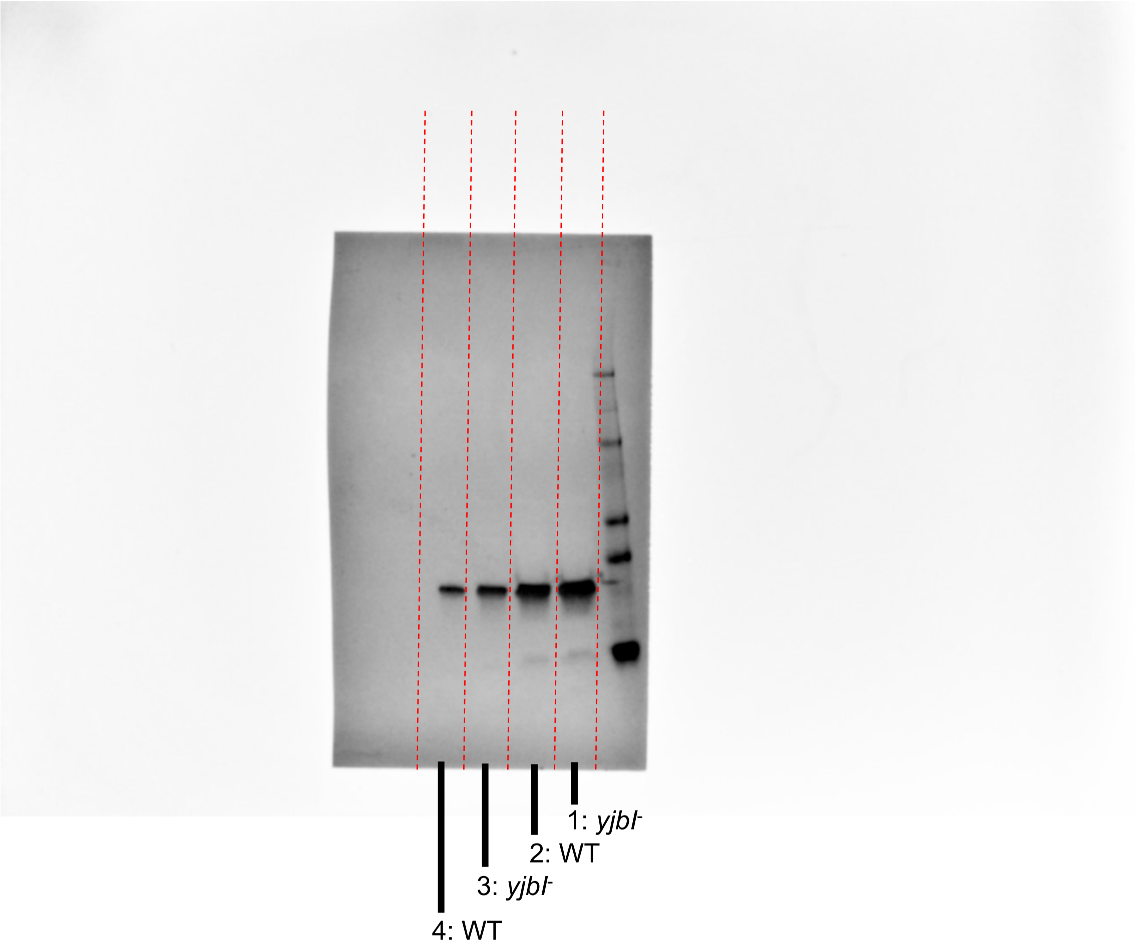

Reproducibility of result with independent insoluble fraction samples

This experiment used same cell samples as the previous soluble fraction samples (No. 1-4).

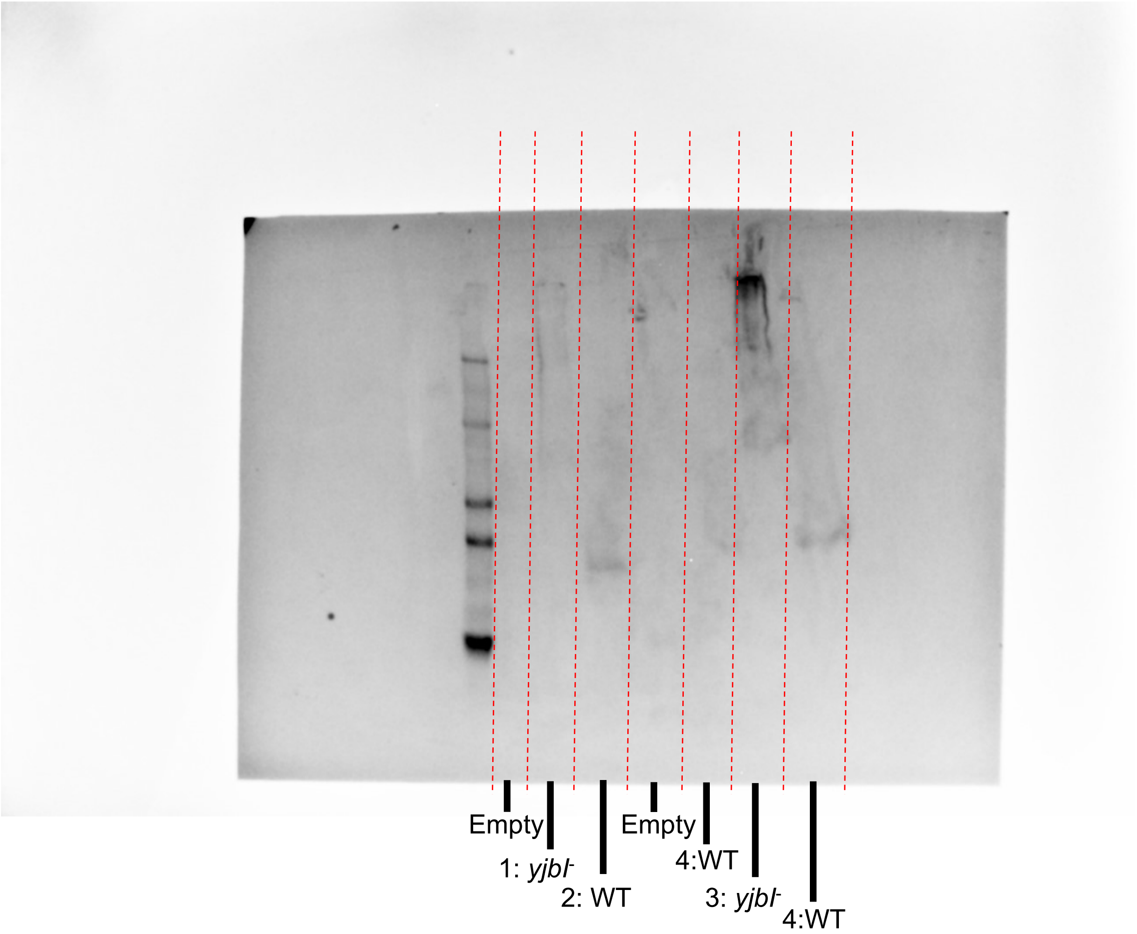

Supplement: Figure 2—source data 1. [file elife-70467-fig2-data1.zip › Figure 2-source data 1/Figure 2-source data 1.pdf]
