# Supplementary material for: Group II truncated haemoglobin YjbI prevents reactive oxygen species-induced protein aggregation in Bacillus subtilis
Source: eLife. 2022 Sep 20;11:e70467. doi: 10.7554/eLife.70467 (PMC9536834; doi:10.7554/eLife.70467)
Supplement: Figure 2—source data 2. [file elife-70467-fig2-data2.zip › Figure 2-source data 2/Figure 2-source data 2.pdf]

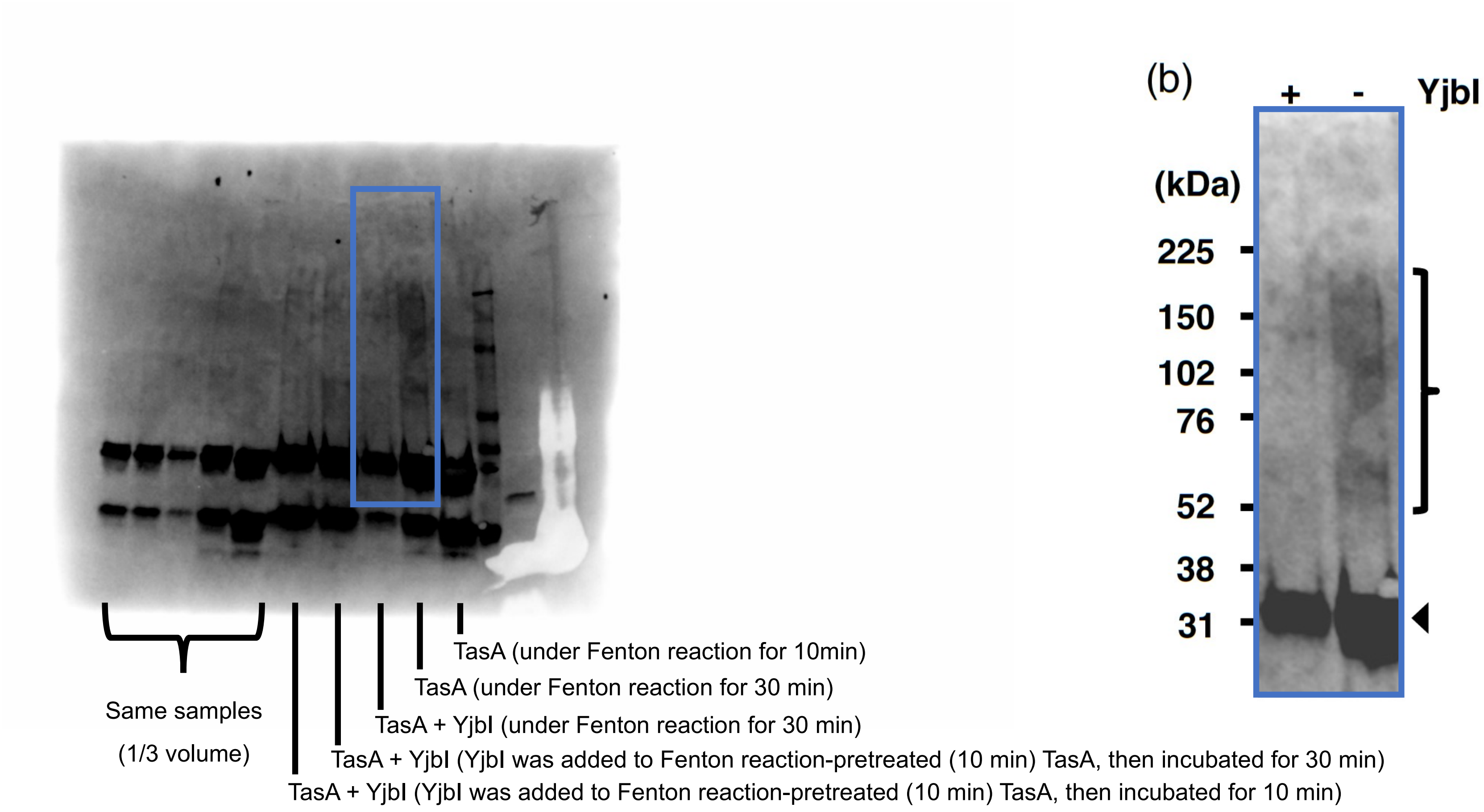
(b)
+
-
YjbI
(kDa)
225
150
102
76
52
38
31
Same samples
(1/3 volume)
TasA (under Fenton reaction for 10min)
TasA (under Fenton reaction for 30 min)
TasA + YjbI (under Fenton reaction for 30 min)
TasA + YjbI (YjbI was added to Fenton reaction-pretreated (10 min) TasA, then incubated for 30 min)
TasA + YjbI (YjbI was added to Fenton reaction-pretreated (10 min) TasA, then incubated for 10 min)
