# Supplementary figures and images for: Group II truncated haemoglobin YjbI prevents reactive oxygen species-induced protein aggregation in Bacillus subtilis
Source: eLife. 2022 Sep 20;11:e70467. doi: 10.7554/eLife.70467 (PMC9536834; doi:10.7554/eLife.70467)

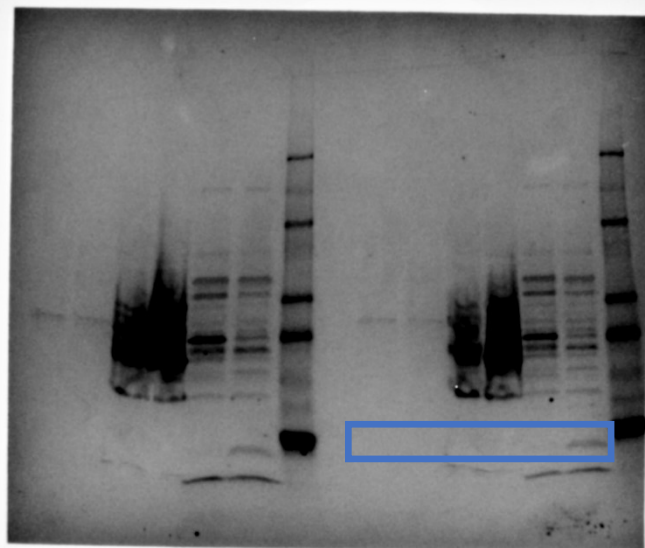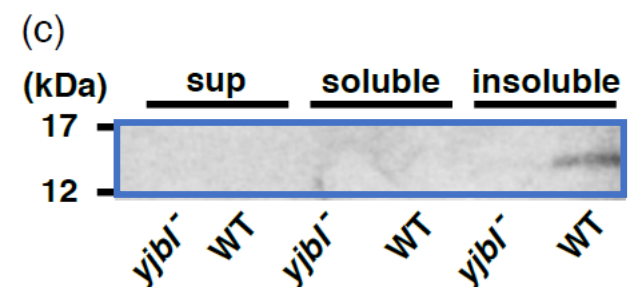

# Property of anti-YjbI antiserum (Western blotting)

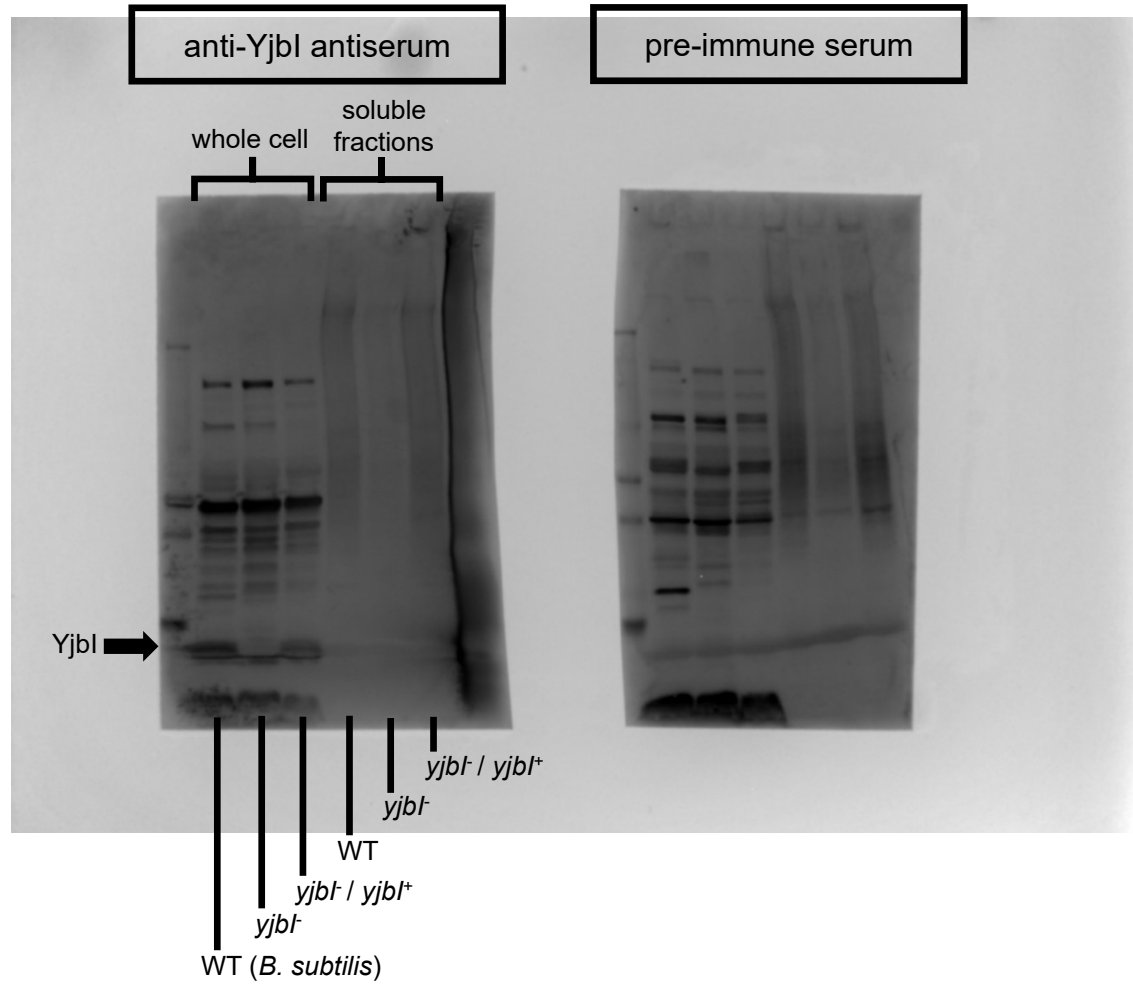

Supplement: Figure 2—source data 3. [file elife-70467-fig2-data3.zip › Figure 2-source data 3/Figure 2-source data 3.pdf]

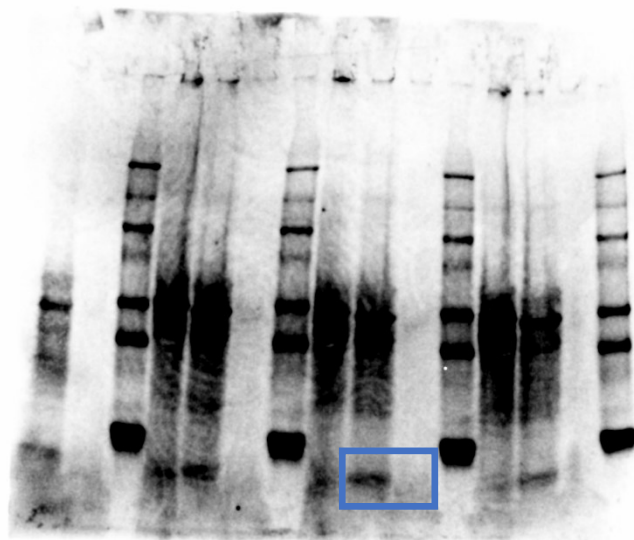

Inversion was applied to the figure.

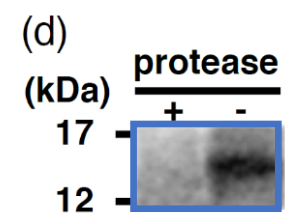

Supplement: Figure 2—source data 4. [file elife-70467-fig2-data4.zip › Figure 2-source data 4/Figure 2-source data 4.pdf]

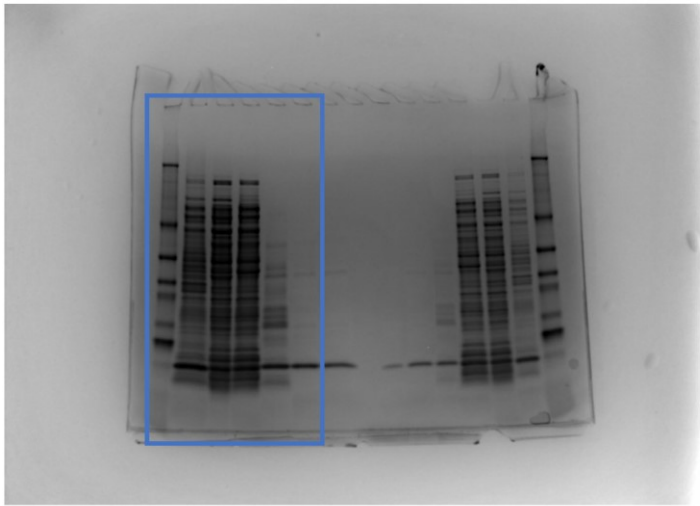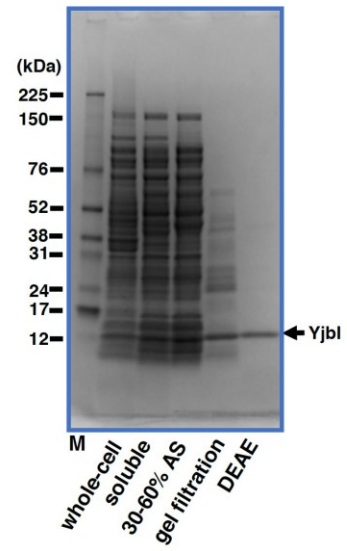

Supplement: Figure 2—figure supplement 1—source data 1. [file elife-70467-fig2-figsupp1-data1.zip › Figure 2-figure supplement 1 source data/Figure 2-figure supplement 1 source data.pdf]

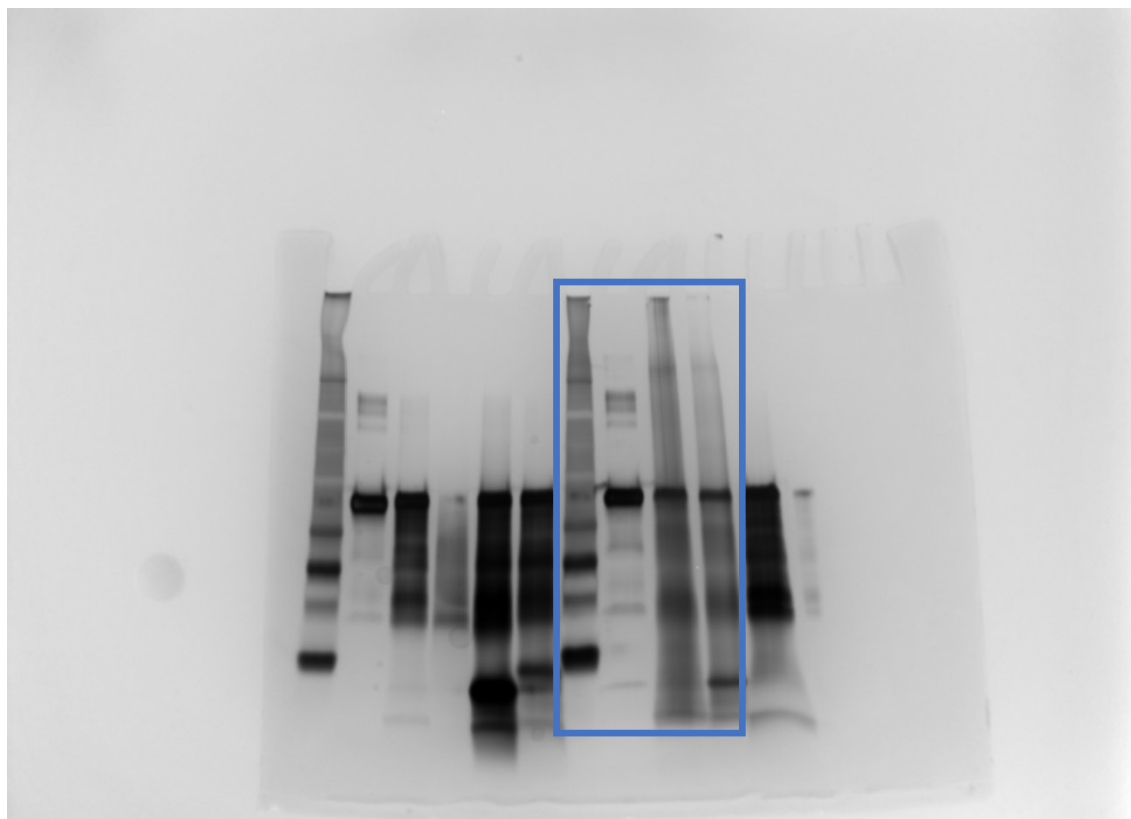

(a)

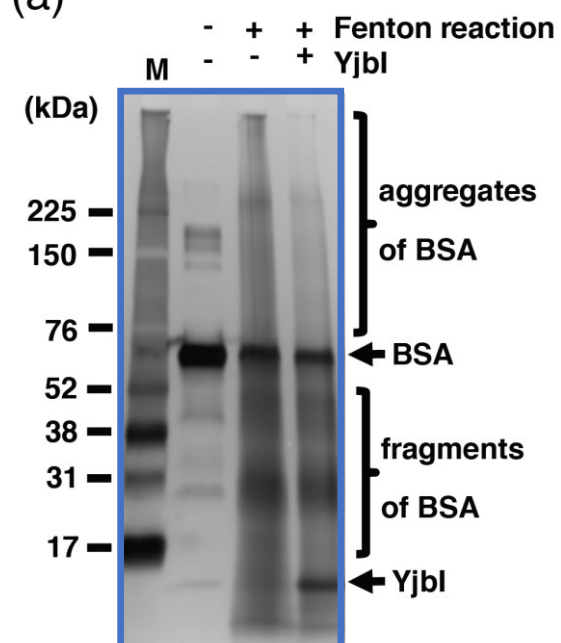

Supplement: Figure 3—source data 1. [file elife-70467-fig3-data1.zip › Figure 3-source data 1/Figure 3-source data 1.pdf]

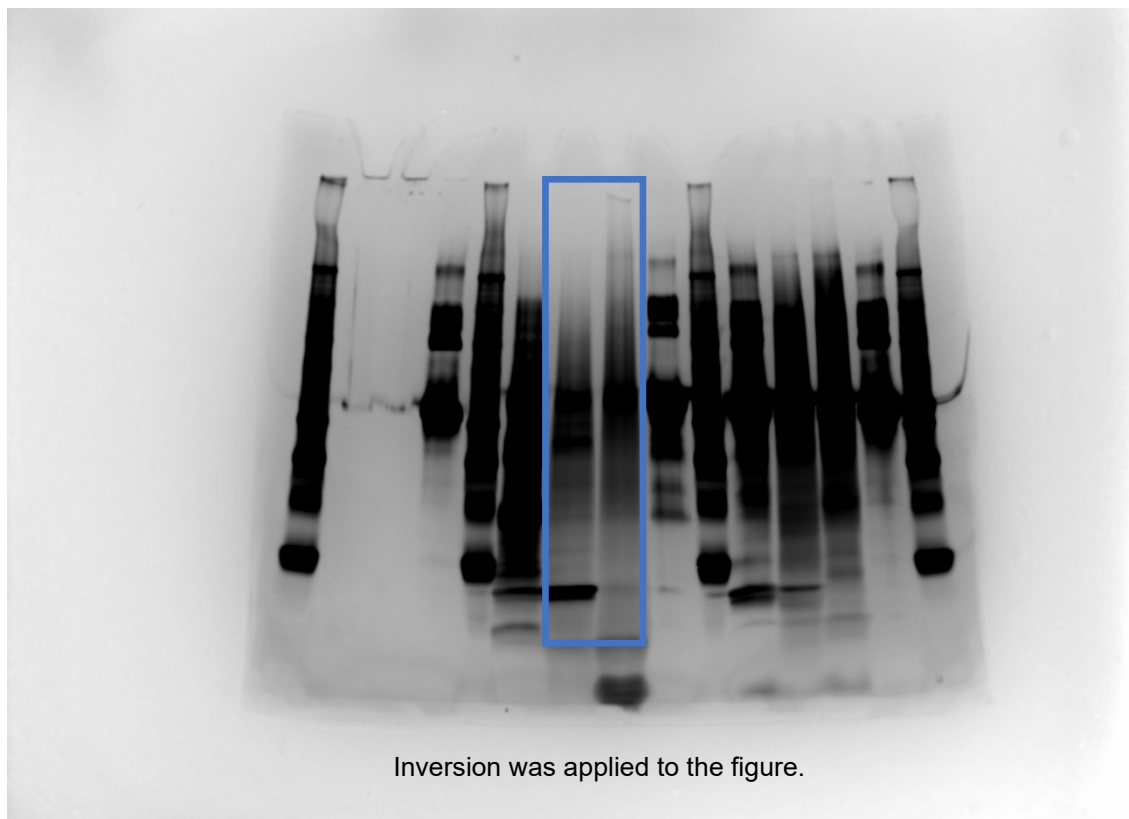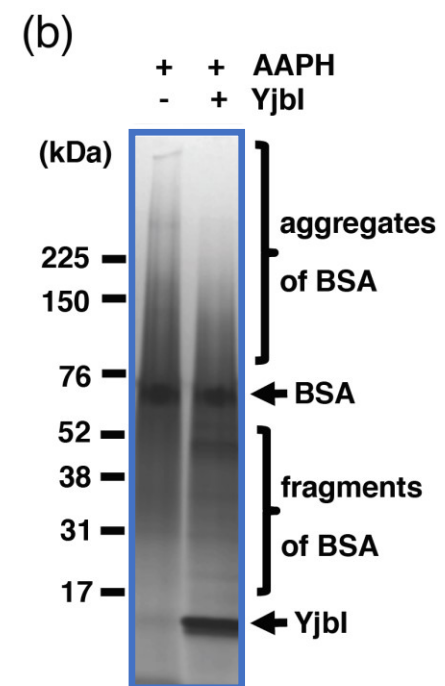

Supplement: Figure 3—source data 2. [file elife-70467-fig3-data2.zip › Figure 3-source data 2/Figure 3-source data 2.pdf]

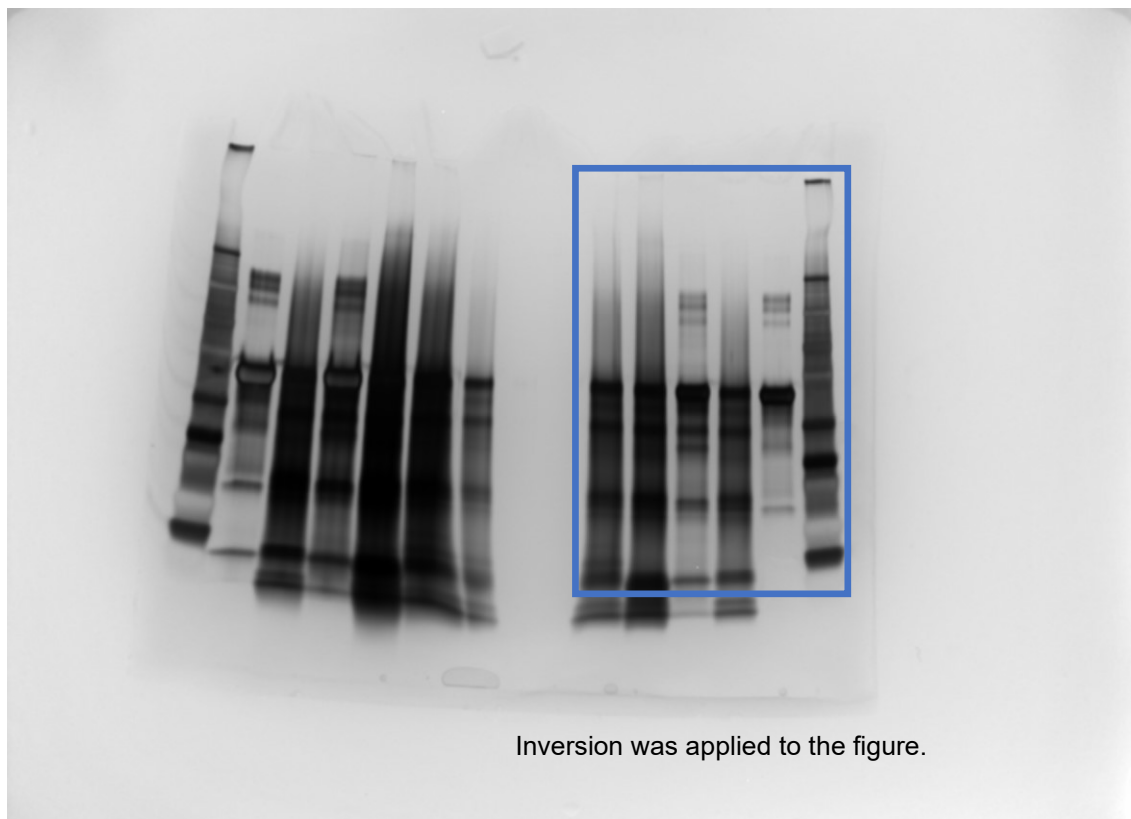

(d)

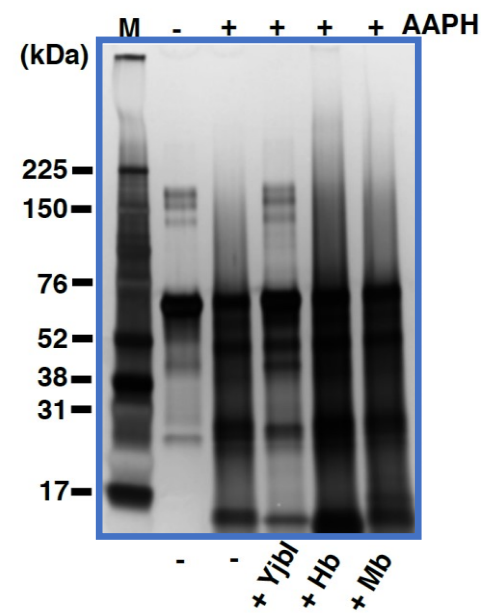

Supplement: Figure 3—source data 3. [file elife-70467-fig3-data3.zip › Figure 3-source data 3/Figure 3-source data 3.pdf]

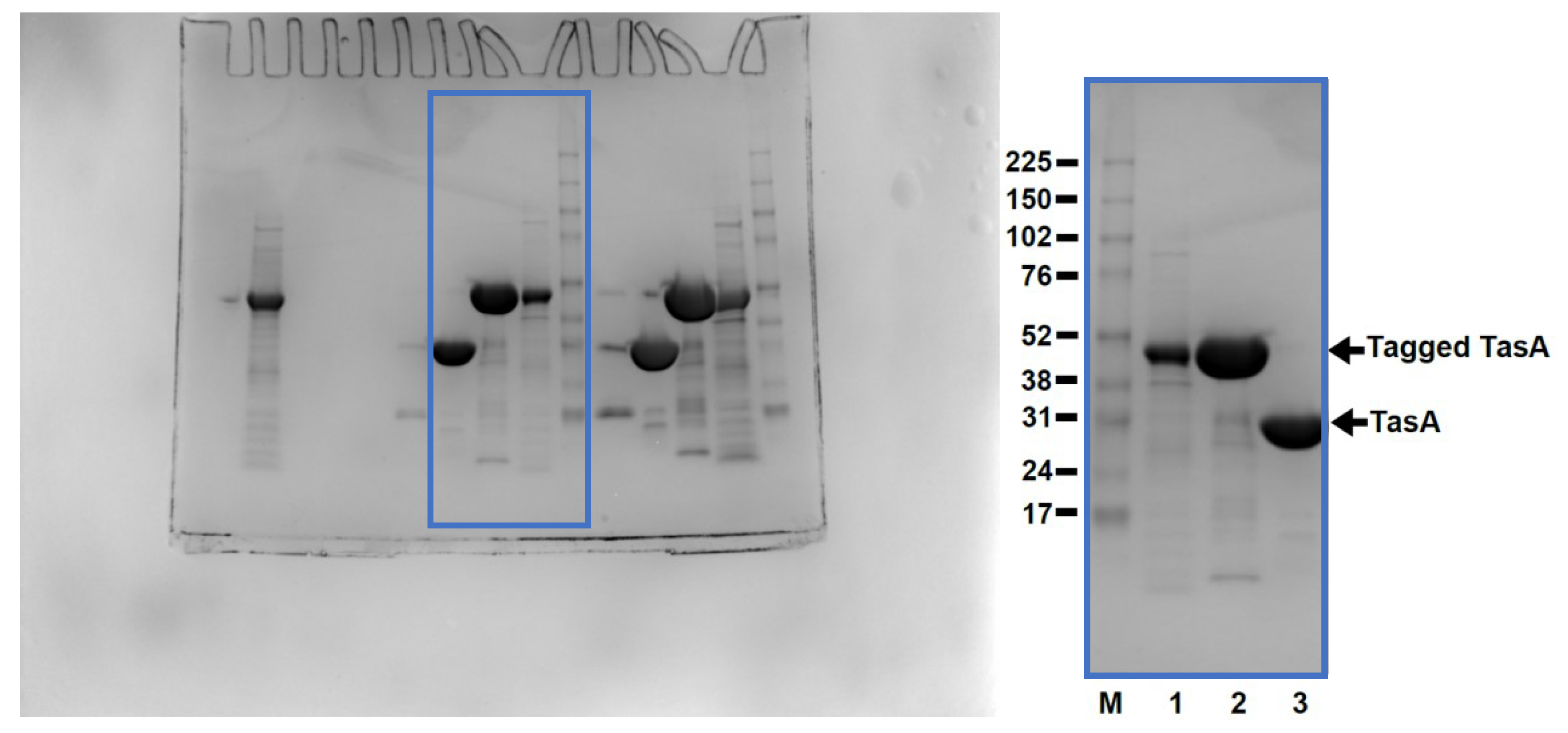

Supplement: Figure 3—figure supplement 1—source data 1. [file elife-70467-fig3-figsupp1-data1.zip › Figure 3-figure supplement 1 source data 1.tif]

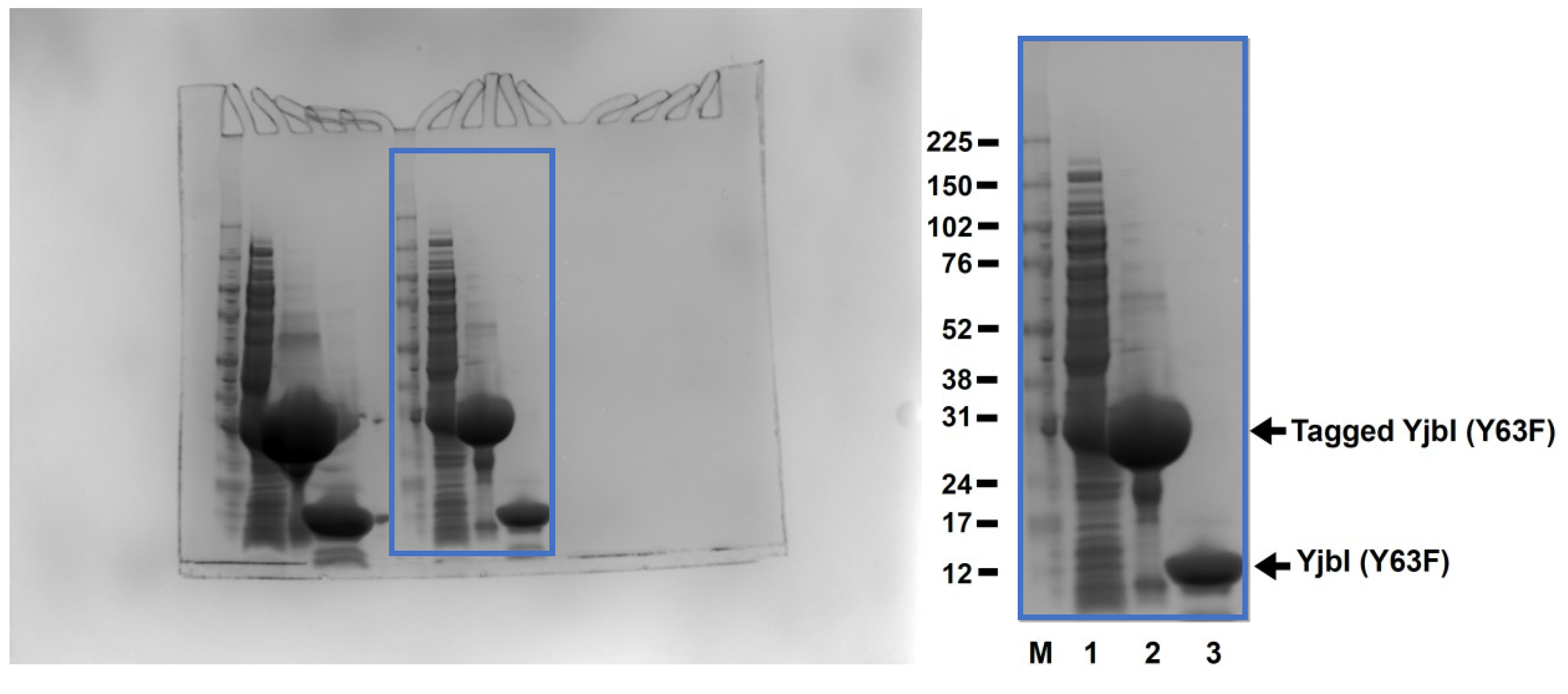

Supplement: Figure 4—figure supplement 1—source data 1. [file elife-70467-fig4-figsupp1-data1.zip › Figure 4-figure supplement 1 source data 1.tif]
